# One year successful outcomes for novel sacroiliac joint arthrodesis system

**DOI:** 10.1186/1750-1164-6-13

**Published:** 2012-12-27

**Authors:** Donald Sachs, Robyn Capobianco

**Affiliations:** 1 Center for Spinal Stenosis and Neurologic Care, PO Box 8815, Lakeland, FL, 33806, USA; 2 SI-BONE Inc, 3055 Olin Ave. Suite 2200, San Jose, CA, 95128, USA

**Keywords:** Minimally invasive, Sacroiliac joint, Arthrodesis

## Abstract

**Background:**

SI joint pain can mimic discogenic low back pain or even radicular pain. Patient presentations vary considerably and conditions may include low back, groin, and/or radicular pain, leading to the potential for inaccurate diagnosis and treatment. Despite the large number of patients with SI joint pain, treatment options have been limited to conservative care involving physical therapy and joint injections, radiofrequency rhizotomy, or traditional open SI joint arthrodesis surgery. The purpose of this retrospective study is to evaluate the safety and effectiveness of MIS SI joint arthrodesis via an ileosacral approach in patients refractory to conservative care.

**Methods:**

We report on the first 11 consecutive patients treated with a novel MIS SI joint fusion system by a single surgeon. Medical charts were reviewed for perioperative metrics and baseline pain scores recorded using a 0-10 numerical rating scale.

**Results:**

Ninety one percent (91%) of patients were female and the average patient age was 65 years (range 45-82). Mean baseline pain score (SD) was 7.9 (± 2.2). Mean pain score at the 12 month follow up interval was 2.3 (±3.1), resulting in an average improvement of 6.2 points from baseline, representing a clinically and statistically significant (p=0.000) improvement. Patient satisfaction was very high with 100% indicating that they would have the same surgery again for the same result.

**Conclusions:**

The results of this small case series illustrate the safety and effectiveness of minimally invasive SI joint fusion using a series of triangular porous plasma coated titanium implants in carefully selected patients. Larger multi centered studies are warranted.

## Background

Low back pain is exceedingly common in modern society, affecting well over 90% of adults at some point in their lives [1]. Apart from the common cold, it is the most common reason for visits to the primary care doctor [1]. Loss of productivity and income combined with medical expenses results in a $60 billion expenditure annually in the US related to low back pain [2]. Treatment of low back pain requires identifying the pain generator(s), which can be a significant challenge due to the multifactorial nature of this condition. Historically, though the sacroiliac (SI) joint was initially suspected as a significant generator of LBP, as more reliably diagnosed conditions such as herniated discs and facet arthropathy became better understood, less focus was placed on the SI joint [3]. Recently there has been a resurgence in consideration of the SI joint as a low back pain generator. Recent published literature reports that 15-30% of patients presenting with low back pain had sacroiliac (SI) joint problems [4]. Additionally, up to 75% of post-lumbar fusion patients will develop significant SI joint degeneration after 5 years [5-7]. SI joint pain can mimic discogenic or radicular low back pain, and patients can present with low back, groin, and/or gluteal pain, leading to the potential for inaccurate diagnosis and treatment [1,8,9].

Despite the large number of patients with SI joint pain, treatment options have been limited to nonoperative care involving physical therapy and joint injections, radiofrequency rhizotomy, or traditional open SI joint arthrodesis surgery. Open arthrodesis procedures reported in the literature require relatively large incisions, significant bone harvesting, and lengthy hospital stays; moreover, they may require non-weight bearing for several months [10-13].

To address these challenges, a novel minimally invasive arthrodesis system has been developed (iFuse Implant System, SI-BONE, Inc. San Jose, CA). The surgical procedure involves placing a series of triangular, porous plasma spray coated titanium implants placed across the target SI joint. The objective of this surgery is to achieve arthrodesis through a permanent linkage across the joint, relying on bone ongrowth for permanent stabilization of the implant.

The purpose of this retrospective study is to evaluate the safety and effectiveness of SI joint arthrodesis via a minimally invasive surgical (MIS) approach in patients refractory to conservative care.

## Methods

We report outcomes of the first consecutive 11 patients treated at a single, community based spine practice between April 2011 and July 2011. Medical charts were reviewed for peri-operative metrics, complications, pain scores (0-10 numerical rating scale) and satisfaction with surgery. As there were no experimental procedures performed in this case series and the data was collected without using personally identifiable information from medical records, no IRB or ethics committee review was required or obtained.

Mean age at the time of surgery was 65 years (range 45-82) and nearly all patients were women (91%) (Table 1). Three patients (27%) had a history of previous lumbar spine surgery: 2 underwent previous lumbar fusion and one had a laminectomy.

**Table 1:** Demographic information

**Patients**	**11**
Age	65 (range 45-82)
Gender	10 F (91%), 1 M (9%)
Prior lumbar spine surgery	3 (27%) 2 fusion, 1 laminectomy

Patients were diagnosed with either degenerative sacroiliitis or sacroiliac joint disruption using a combination of history, clinical exam, and positive diagnostic injection. All patients presented with low back and SI joint pain. In addition, 82% also complained of buttock pain. All patients had failed at minimum 6 months of nonoperative care consisting of a combination of medication optimization, physical therapy and lumbar spine injections. A thorough physical and clinical exam was performed on all patients in order to correctly determine the pain generator. Provocative physical examination maneuvers were used to guide subsequent diagnostic activities. Radiographic studies were performed to rule out pathology in the lumbar spine and hip. When clinical, physical and radiographic examinations were concordant, patients were sent for confirmatory image-guided diagnostic injections of the SI joint. A 75% reduction in pain immediately following injection of local anesthestic was used to confirm the SI joint as the pain generator [7].

Minimally invasive SI joint fusion using the iFuse Implant System (SI-BONE Inc., San Jose, CA) was performed in all cases by a single neurosurgeon.

### Technique description

**Figure 1:**
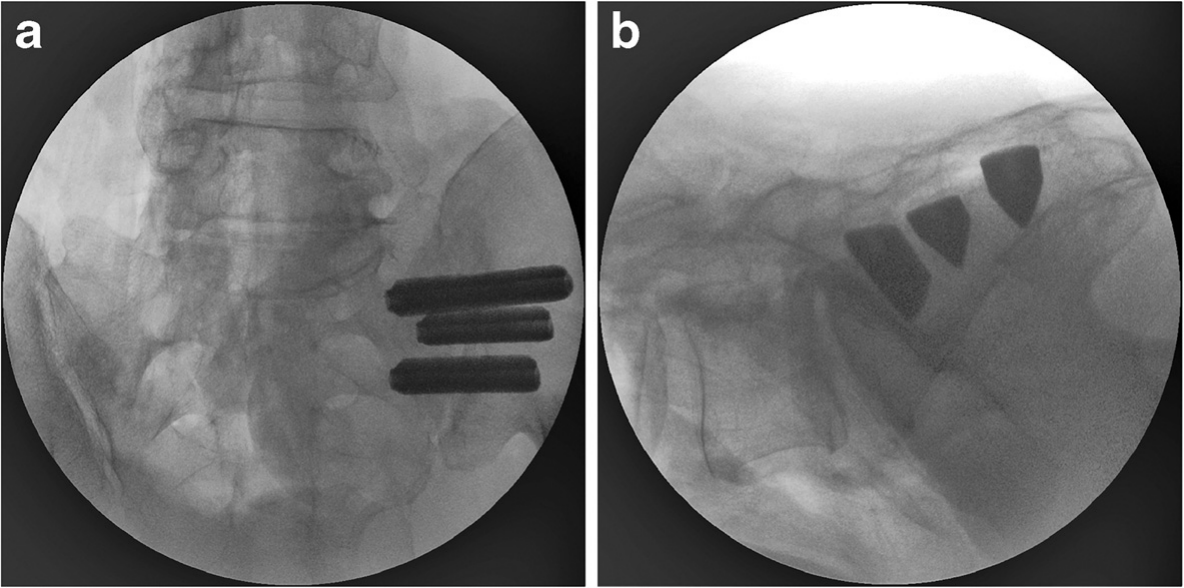
a) AP and b) lateral view of all three implants in place.

The procedure is performed by placing a series of 3 triangular shaped porous plasma coated titanium implants across the SI joint using intermittent fluoroscopy to monitor instrument and implant placement (Figure 1). General endotracheal anesthesia is administered and the patient is placed in the prone position on a radiolucent table. After the lateral buttock and pelvis are prepped in the normal sterile fashion, a 3cm skin incision is made. Through this small incision, the gluteal fascia is penetrated bluntly and the muscle is split longitudinally to gain access to the outer table of the ilium. A Steinmann pin is passed through the ilium across the SI joint in to the center of the sacrum, lateral to the neural foramen. A soft tissue protector is inserted over the pin and a drill is used to create a pathway and decorticate bone through the ilium to the sacrum. After the drill is removed, a broach is malleted across the joint where autologous bone graft is harvested. Finally, the implant is malleted into place. The cephalad implant is routinely placed within the sacral ala. A pin-guide system is used to facilitate placement of the subsequent implants. The second implant is generally located above or adjacent to the S1 foramen and the third between the S1 and S2 foramen. The incision is then irrigated and the tissue layers are closed with Vicryl and Monocryl. Patients are instructed to ambulate with the assistance of a walker for the first 4 weeks after which time toe touch ambulation is recommended for another 4 weeks. After 8 weeks of gradual return to full weight bearing, patients begin 4 weeks of physical therapy. Patients are routinely followed in the clinic at one week, six weeks and 3 months post-operatively.

### Outcomes

Pain related to the SI joint was assessed pre-operatively and post-operatively at 12 months per standard procedure at our practice. Patients were asked to rate their pain using a 0-10 numerical rating scale with 0 representing no pain and 10 representing the worst pain imaginable. Satisfaction was assessed by asking the patient if s/he would have the same surgery again for the same outcome.

## Results

We report on 11 consecutive patients who were treated with minimally invasive SI joint fusion. A total of 12 SI joints were treated: 8 right- and 4 left-sided. Average (± SD) operating time was 78 ± 32 minutes and blood loss was less than 50cc in all cases (Table 2). No intraoperative complications were observed. At one year, there were no surgical revisions.

**Table 2:** Peri-operative characteristics

**Joints treated**	**12**
Right SI joint	8
Left SI joint	4
Staged bilateral surgery	1
OR time (min)	77.5 ± 31.8
Estimated blood loss (cc)	21.8 ± 18.9

### Clinical outcomes

Mean baseline pain score (SD) was 7.9 (± 2.2). Mean pain score at the 12 month follow up interval was 2.3 (±3.1). The mean (SD) change in pain score was 5.4 (3.4) points (p<.000). A clinically significant benefit, defined as a >2 point change from baseline, was observed in 8 (80%) patients [14]. Despite 2 patients not reaching the threshold of change in pain score deemed clinically relevant, 100% of patients expressed satisfaction with the procedure, indicating that they would have the same surgery again for the same result. One patient developed mild piriformis syndrome that resolved with physical therapy. One patient developed low back pain and was sent for facet injections, which were effective in relieving pain.

## Discussion

SI joint symptoms can present as pain in the SI joint, low back, hip, groin, or buttock. As a result, a careful and thorough clinical and physical exam must be performed to correctly identify the pain generator. Positive provocative physical examination maneuvers combined with 75% pain relief after imaged guided SI joint injection is a reliable method for diagnosing the SI joint as the pain generator [15,16].

Recent reports of MIS approaches to SI joint arthrodesis using screws show relatively good clinical results with room for improvement in outcomes and technique. Al-Khayer et al. reported on 9 patients using a single hollow modular anchorage (HMA) screw packed with bone graft [17]. All patients experienced a clinically significant improvement in VAS pain scores and all but 1 patient improved in function as measured by ODI. One patient suffered a deep wound infection. Khurana at al. also report on HMA screws with demineralized bone matrix in a cohort of 15 patients with relatively good outcomes [18]. Wise and Dall reported on 13 patients and 19 joints using 11×25 mm threaded fusion cages packed with rhBMP-2 with good clinical results. The cage was inserted posteriorly within the joint rather than bridging the joint as reported in previous studies. Due to the off label nature and elevated cost associated with rhBMP-2, autologous iliac crest harvest was suggested. However, studies suggest that this can lead to further degeneration of the SI joint [10]. Although short term outcomes are favorable, these techniques do not address common issues observed with orthopedic screws such as loosening and breakage [19].

Advantages of this reported MIS SI joint fusion implant technique include a small incision, relatively short operating time, minimal blood loss, bone and ligament preservation, and a relatively short period of immobilization. The triangular shape combined with an interference fit of the titanium implant used in this cohort was designed to minimize rotation, micromotion and avoid issues encountered with traditional screws. In our cohort of patients undergoing MIS SI joint fusion, clinical outcomes were favorable with 80% of patients experiencing a clinically significant benefit at 12-months. One patient did not report improvement in pain. This 72 year old female patient presented with a 20 year history of low back and SI joint pain with multiple back surgeries. She felt she was unable to distinguish SI pain from low back pain, which may have confounded the results of SI joint surgery. The other patient with previous back surgery had a substantial improvement in pain. Though this represents the results of two patients, one cannot assume any relationship between prior back surgery and clinical improvement after SI joint surgery.

As with all surgical procedures, there are inherent risks. Possible risks of the reported procedure include implant malposition, nerve damage, loosening of implants, and failure to relieve symptoms. These risks are minimized with procedural training, implant design features and proper patient selection. Implant costs may be considered a disadvantage on the surface, however relief from chronic debilitating pain has the potential to save on disability related expenditures. Proper patient selection cannot be overemphasized to ensure a proper diagnosis and treatment plan in low back pain patients.

Although our study sample size is small, the results of minimally invasive SI joint surgery appear promising. All patients presented with low back and SI joint pain. Favorable outcomes in these patients underscore the necessity to suspect the SI joint as a pain generator in patients with low back pain. Special attention should be paid to the SI joint after lumbar spine surgery to avoid the potential for inaccurate diagnosis and treatment. Furthermore, this minimally invasive approach significantly benefits the elderly population, who are not candidates for other conventional techniques due to poor bone quality, delayed healing and reduced mobility. This segment of the population is not likely to respond well to physical therapy alone in part because of the degenerative nature of SI joint disease. The MIS procedure described herein affords this segment of the population an opportunity to regain mobility, alleviate SI joint and low back pain caused by SI joint issues and experience an improved quality of life.

## Conclusion

When conservative measures fail, minimally invasive SI joint fusion using a series of triangular porous plasma coated titanium implants is a safe and effective treatment option in carefully selected patients.

## Competing interests

Donald Sachs: Consulting agreement with SI-Bone Inc., no stock or royalties. Robyn Capobianco: Part time clinical affairs employee at SI-Bone, Inc.

## Authors’ contributions

DS performed all surgical procedures, performed data collection and interpretation and critical review of manuscript. RC was involved in drafting manuscript and data analysis. Both authors read and approved the final manuscript.
